# Training refugee and asylum-seeking doctors: a cohort study of the UK REACHE programme

**DOI:** 10.1136/bmjopen-2025-105550

**Published:** 2025-11-04

**Authors:** Aaron Drovandi, Samuel Barrett, Jouher Kallingal, Aisha Awan

**Affiliations:** 1 School of Medical Sciences, The University of Manchester, Manchester, UK; 2 Refugee & Asylum Seekers Centre for Healthcare Professionals Education, Salford, UK; 3 Northern Care Alliance NHS Foundation Trust, Salford, UK; 4 The University of Manchester School of Medical Sciences, Manchester, UK; 5 Department of Medical Education, University of Manchester, Manchester, UK

**Keywords:** Education, Medical, Refugees, Health Workforce

## Abstract

**Abstract:**

**Objective:**

Refugee and asylum-seeking (RAS) doctors benefit from specialised support to achieve medical registration, though there is limited published evidence from programmes supporting them. This study describes the outcomes of the Refugee and Asylum Seekers Centre for Healthcare Professionals Education (REACHE), a UK-based comprehensive language, clinical and professionalism skills training programme in supporting RAS doctors.

**Design:**

Prospective cohort study.

**Setting:**

Single educational centre.

**Participants:**

607 doctor learners admitted to the REACHE programme.

**Primary and secondary outcome measures:**

Learner characteristics, demographics and learning journeys (including duration studying and examination pass rates) and alumni outcomes (including registration, specialties obtained and practice locations).

**Results:**

Of 607 doctor learners having entered the programme, 109 are currently on the programme and 498 are alumni. Learners took a median 1.3 years between arriving in the UK and contacting REACHE, with a median 6.4 years of prior clinical experience. Learners had high first-attempt pass rates (≥85%) for occupational language and clinical examinations required for registration. Of the alumni, 258 (51.8%) completed the programme (median time of 2.1 years) and achieved registration for practice and National Health Service employment. Of those who left before completion, who had access to 10 year post-programme scaffolded support, nearly one quarter (53 of 228; 23.2%) also achieved registration. 82 alumni are on specialty registers.

**Conclusion:**

Retraining programmes such as REACHE can effectively support RAS doctor requalification, providing the UK medical workforce with experienced professionals. Improved referral pathways, sustainable funding and incorporation into government health workforce strategies are expected to strengthen already substantial achievements of programmes such as REACHE.

**STRENGTHS AND LIMITATIONS OF THIS STUDY**

- Data were sourced from reliable internal and external databases.
- The large sample size and diversity of participants support generalisability of the findings across refugee doctors from any background.
- Participants may be those with higher capacity to invest time in learning and securing registration and employment compared with the broader refugee and asylum seeker population.
- Examination pass rate accuracy may have been affected by participants attempting examinations without disclosing to the programme team.

## Introduction

 The displacement of people from their home countries due to geopolitical issues including war, famine, the climate crisis and persecution has been a longstanding global issue.^1^ Refugees and asylum seekers (RAS) often enter host countries with little or no financial stability, job prospects or support networks and require comprehensive support systems to allow their integration into new communities and lead meaningful lives.^2 3^ Within the UK, there are over 230 000 refugees and 125 000 pending asylum seekers, often placed in UK Home Office accommodation and at risk of sudden homelessness due to shifting governmental policies.^1 4^ Many RAS were experienced healthcare professionals (HCPs) in their country of origin, and after relocation, they became increasingly deskilled and faced losing their professional identity by needing to take on jobs not commensurate with their formal training and clinical experiences.^5^

Concurrently, there are global health workforce shortages; theWHO forecasts that by 2035 there will be a shortage of 12.9 million HCPs.^6^ Within the UK, despite nearly one-eighth of the total workforce being employed in healthcare, there were over 100 000 healthcare-related vacancies in England alone in 2023.^7 8^ Reasons behind these shortages include the global ageing population and more complex requirements for care within communities and workforce migration out of healthcare or into healthcare jobs in countries offering better working conditions.^9 10^ These shortages reduce patient access to care and cause longer wait times, delayed diagnosis and ultimately poorer health outcomes.^11^

RAS HCPs entering the UK are unable to practise their profession. Restrictions result in their working in support roles such as healthcare assistants or phlebotomists, often requiring additional training, despite their existing expertise and qualifications. Educational programmes tailored towards RAS HCPs support this vulnerable population in re-entering positions aligned with their skills and training and facilitate their transition from reliance on state funds to supporting themselves and their dependents, while alleviating health workforce shortages.^5^ Such programmes are ethical, socially responsible and financially viable, through alleviating workforce shortages without depleting the Global South of their own workforce and improving quality of life and mental health of this vulnerable population.^12 13^

These educational programmes require sustainable funding, which in turn requires evidence of benefit, with limited data currently available on existing services and how they benefit the RAS HCP and their host countries.^14^ Within the UK, ‘The Refugee and Asylum Seekers Centre for Healthcare Professionals Education’ (REACHE) is the largest RAS HCP training programme, operating since 2003 to provide specialist occupational language education, pastoral care, acculturation to the National Health Service (NHS) and UK life, clinical training and placements to guide learners through to professional registration and secure clinical work.

**Figure 1:**
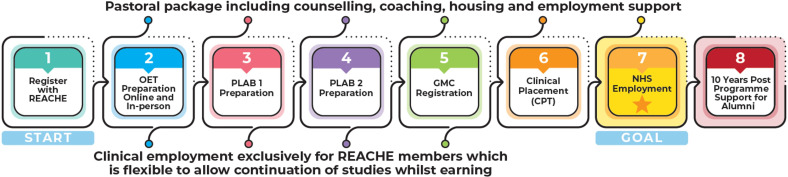
Illustration of the REACHE training programme. CPT, Curricular Practical Training; GMC, General Medical Council; OET, Occupational English Test; NHS, National Health Service; PLAB, Professional and Linguistic Assessments Board; REACHE, Refugee and Asylum Seekers Centre for Healthcare Professionals Education.

Figure 1 illustrates the individual components and directionality of learner journeys through the programme. On entry into the programme, learners are provided with continual pastoral support on a range of personal issues that support settling into the UK, as well as support in securing ‘lower-grade’ clinical employment for improved financial stability while learning. The educational programme is run across three semesters in a hybrid format, taught and supported by clinical tutors and senior lecturers from the University of Manchester and language teachers at a hospital-based education centre. English-language training and exam preparation are provided at two levels according to English proficiency at entry, including practice conversations and other short activities, daily questions and practice examinations. The clinical curriculum is based on the requirements for the General Medical Council (GMC) Medical Licensing Exam. Learners also complete a reflective portfolio, attend regular whole day ‘integrated communication and clinical skills’ days covering topics to help adapt to life and work in the UK, clinical role plays, clinical reasoning, opportunities for research and audit and simulation suite sessions alongside their weekday teaching.

REACHE learners (most of whom are medical doctors) have unique educational requirements in addition to having potentially challenging personal circumstances while managing traumas associated with displacement. On achieving registration, RAS doctors may work clinically in the UK, entering at a ‘Foundation Year 2’ level, though this may not be at their highest level of qualification. The aim of this study was to demonstrate the outputs of the REACHE programme in supporting RAS doctors in securing clinical employment within the UK and serve as an example to neighbouring nations on the value of RAS doctor training.

## Methods

This study is reported according to the STrengthening the Reporting of OBservational studies in Epidemiology criteria.^15^ Ethical approval was granted by the University of Manchester Proportionate Research Ethics Committee (2024-19920-35769).

### Study design and participants

This was a retrospective analysis of prospectively collected data of RAS doctors admitted to the REACHE programme (based at the Salford Royal Hospital, UK) from programme establishment (2003) to December 2024. All RAS HCPs residing in the UK with basic English language skills can apply to become a REACHE learner, and if accepted, attend hospital setting-based language and training and receive support as illustrated in figure 1. For this study, all RAS doctors admitted to the REACHE programme were eligible for inclusion, including those who did not complete the programme and achieve professional registration. RAS doctors excluded were those that partially completed the admission process but did not formally begin learning. All REACHE learners provide consent to have their data stored in the REACHE database and for anonymised data to be used for research and grant funding purposes.

### Data sources and data collection

There were three sources of data for this study. First, on admission to the REACHE programme, RAS doctors provide details of their personal and professional circumstances. Personal details included age, gender, birth country, languages spoken, disabilities, family situation and legal status for entry to the UK. Professional details included profession and any specialties, degrees awarded, years of experience, duration since last working clinically and any non-clinical work since arriving in the UK. Second, the REACHE database captures learner journeys through the REACHE programme, such as duration within the programme and specific strands of the programme, examination attempts and pass rates, and clinical attachments and clinical placements attended. Third, the GMC, the regulator for doctors in the UK, provides publicly accessible details of registered doctors such as date registered, practice location, any limitations to practice and any specialties.

### Data analysis

Data were extracted from the REACHE database and regulator register lists and coded numerically for import into SPSS v29 (IBM Corp, Armonk, NY, USA) for analysis. Learner characteristics, learning journeys and employment details were summarised narratively. Learning journeys and outcomes (duration to complete, examination pass rate and achieving registration) were compared between learner age, gender, entry pathway into the UK, having dependents, years of previous clinical experience, duration since working clinically, having a postgraduate degree and having acquired a specialty. Binary logistic regression, linear regression, independent sample t-tests and X^2^ tests were used according to the relevant dependent and independent variables. It was also planned to compare specific employment outcomes according to these characteristics, though missing data from alumni prevented this. Locations of practice of alumni from the registrant database were inserted into Power BI to generate illustrations of alumni practice locations in the UK.

## Results

### Entry to programme

From inception (April 2003) to 31 December 2024, 788 learners were admitted to the REACHE programme. Of these, 607 (77.0%) were doctors (the cohort of interest in this study) and 181 (23.0%) other professions (including nurses, pharmacists, dentists and radiographers). The median time for doctor learners from entering the UK to initial contact with REACHE was 1.3 years (IQR of 6 months to 3 years, a minimum of 2 weeks and maximum of 15.6 years), with word of mouth being the most frequent method for hearing about REACHE (263; 43%), followed by online searches (71; 12%) and direction from UK medical associations (eg, British Medical Association or the GMC, 68; 11%) or refugee or charity agencies (59; 10%).

Table 1 summarises the characteristics of these 607 RAS doctors. In summary, two-thirds (410; 67.5%) were male, with a mean age of 36 years, with Iraq, Syria and Sudan being the most common countries of origin. Nearly half (259; 42.7%) had dependents. Learners had an average of 8.2 years clinical experience before entering the UK, with one-third (212; 34.9%) being clinical specialists in their home country, and more than 10% having postgraduate degrees, including 47 (7.7%) with a master’s degree and 14 (2.3%) with a doctoral degree (PhD).

**Table 1:** Characteristics of doctors attending Refugee and Asylum Seekers Centre for Healthcare Professionals Education (n=607)

Characteristic	Values
Gender	
Male	410 (67.5%)
Female	197 (32.5%)
Age (on entry to REACHE)	
Mean (SD)	36 (7.2)
Median (IQR)	35 (31–41)
Range	24–58
Country fleeing*	
Afghanistan	56 (9.2%)
Democratic Republic of the Congo	15 (2.5%)
Egypt	15 (2.5%)
Iran	31 (5.1%)
Iraq	104 (17.1%)
Libya	47 (7.7%)
Pakistan	22 (3.6%)
Sudan	74 (12.2%)
Syria	77 (12.7%)
Ukraine	18 (3.0%)
Entry pathway	
Refugee	343 (56.5%)
Asylum seeker	112 (18.5%)
Other	138 (22.7%)
Children	
None	348 (57.3%)
One	81 (13.3%)
Two	90 (14.8%)
Three	44 (7.2%)
Four or more	44 (7.2%)
Years clinical experience†	
Mean (SD)	8.2 (6.8)
Median (IQR)	6.4 (3.0–11.5)
Range	0–37
Advanced degrees	
None stated	537 (88.5%)
Diploma	9 (1.5%)
Masters	47 (7.7%)
PhD	14 (2.3%)
Specialty‡	
None	395 (65.1%)
General internal medicine	17 (2.8%)
General practice	28 (4.6%)
Obstetrics and gynaecology	25 (4.1%)
Paediatrics	22 (3.6%)
Anaesthetics	11 (1.8%)

Data are presented as number (percentage), mean (SD) or median (IQR).

* There are over 55 countries learners had to flee from, with the ten most common countries included, and the full list available in online supplemental appendix 1.

† Years of experience calculated by subtracting their UK arrival date from their degree award date.

‡ There are over 35 specialties present across learners, with the five most common specialties included.

REACHE, Refugee and Asylum Seekers Centre for Healthcare Professionals Education.

### Programme journey

**Figure 2:**
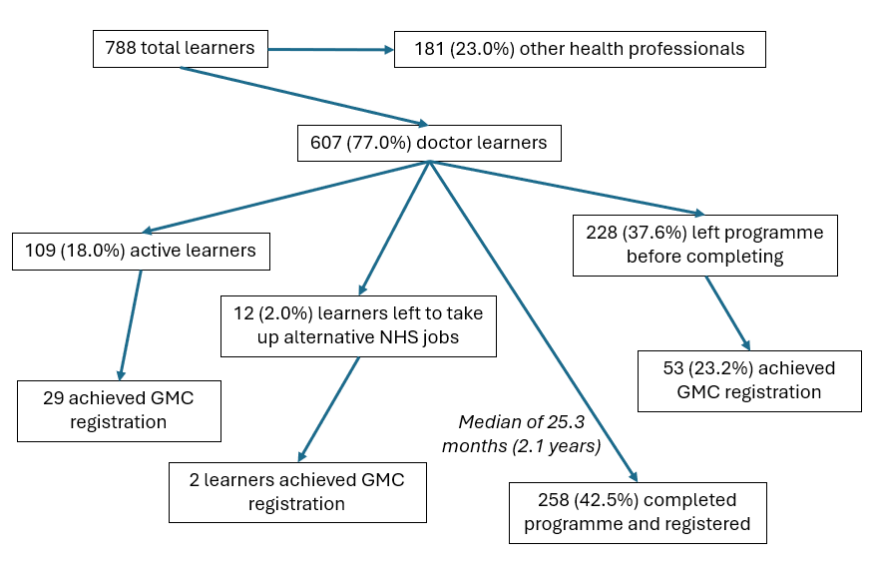
Flow diagram of the Refugee and Asylum Seekers Centre for Healthcare Professionals Education learners over the past 22 years. GMC, General Medical Council; NHS, National Health Service.

As of the end of 2024, 109 (18.0%) of the 607 doctor learners are actively progressing through the programme, and 498 (82.0%) are alumni. Of those having left the programme, 12 (2.0%) took up alternative posts within the NHS, 258 (42.5%) formally completed the programme and 228 (37.6%) left before completing the programme formally (see figure 2). There were several recorded reasons for leaving the programme early, with common issues cited including forced or voluntary relocation elsewhere in the UK, issues with the UK Home Office and their refugee or asylum-seeker status, bereavement, competing non-clinical working requirements (such as reporting to the Department for Work Pensions), personal health issues, non-attendance of classes or becoming non-responsive to communications (lost to follow-up).

Passing two types of examination is required prior to achieving GMC registration in the UK. First, an English-language examination, either the International English Language Testing System (IELTS; the accepted test of registration bodies prior to 2018) or the Occupational English Test (OET; accepted by the GMC from February 2018). Second, the Professional and Linguistic Assessments Board (PLAB) test, composed of a 180 multiple-choice questionnaire (referred to as the applied knowledge test or ‘PLAB 1’), and a 16-stage clinical scenario examination (referred to as the clinical and professional skills assessment or ‘PLAB 2’). From the 284 learners attempting the IELTS, the first attempt pass rate was 65.5% (186 out of 284). For the 142 learners attempting the OET (becoming the English-language test used by REACHE from 2018 onwards), the first attempt pass rate was 88.0% (125 out of 142). For the 311 learners attempting PLAB 1, the first attempt pass rate was 90.7% (282 out of 311), and for 277 learners attempting PLAB 2, the first attempt pass rate was 90.6% (251 out of 277). The average duration learners took to complete the REACHE programme was 26.8 months (SD 18.9 months) and a median of 25.3 months. This includes induction into the programme, attending OET classes and passing the OET exam, attending clinical classes and passing PLAB 1 and PLAB 2, attending professionalism classes, preparing for work and interview skills sessions, achieving GMC registration, going on a 3 to 6 month clinical placement and securing employment within the NHS (see figure 1).

Some examination pass rates were significantly associated with certain learner characteristics. Female learners were significantly less likely to pass iIELTS on the first attempt compared with male learners (43.6% vs 64.1%, p=0.002), with the opposite trend for PLAB 2 (96.4% vs 88.1%, p=0.031). Those with a specialty were also less likely to pass IELTS on their first attempt compared with those who did not (49.0% vs 63.9%, p=0.014). There were no significant associations between age, entry pathway, clinical experience, having children, duration since last practising clinically, having a postgraduate degree with OET or PLAB 1 examination success and the duration to complete the REACHE programme.

### Post-programme registration and employment

As of the end of 2024, 313 REACHE alumni (68.3% of 498) have registered with the GMC, including those who formally completed the programme and registered as part of their programme journey and those who registered after leaving the programme. Many of these were able to use signposting and resources shared by REACHE after leaving the programme before official completion, supporting those exiting early still being able to gain GMC registration. 82 (13.5%) of REACHE learners (26.2% of alumni) are listed on the general practitioner or specialty registers, 13 of which had two specialties and one with three specialties. The number of specific specialties is listed in online supplemental appendix 1, notably including 50 general practitioners and 11 general internal medicine specialists. Six of these 50 general practitioners registered without formally completing the REACHE programme, as did one specialist, which can occur when a learner has secured a job prior to commencing or completing the clinical placement element of the REACHE programme (see figure 1).

The likelihood of achieving professional registration was significantly associated with three of the independent variables: age, entry pathway and clinical experience. Older learners had a decreased likelihood of registering (OR 0.966, 95% CI 0.944 to 0.988, p=0.003) with each year of age associated with a 3.4% reduction in likelihood of achieving registration. Increased clinical experience had the same trend, with each additional year of clinical experience associated with a 4.1% reduction in likelihood of achieving registration (OR 0.959, 95% CI 0.935 to 0.983). Asylum seekers were also significantly less likely to achieve registration compared with refugees (36.6% vs 57.7%, p<0.001).

**Figure 3:**
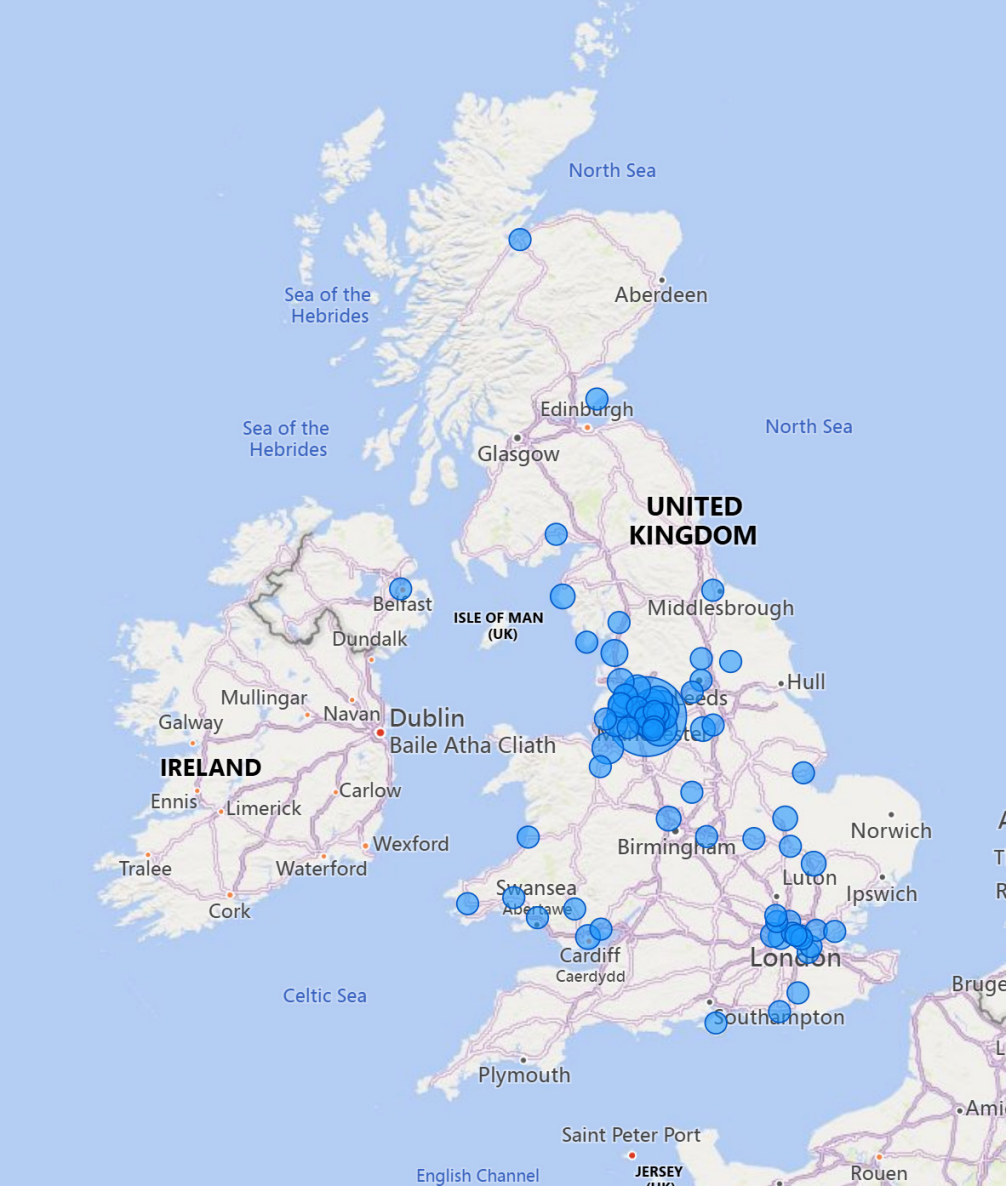
Illustration of practice locations of Refugee and Asylum Seekers Centre for Healthcare Professionals Education alumni.

The location of practice is illustrated in figure 3 (and tabulated in online supplemental appendix 3), where most alumni remain in the Greater Manchester area where REACHE is based. The London area is also a common practice location for alumni, with several other locations throughout the North and Midlands of the UK also employing REACHE doctor alumni.

## Discussion

This cohort analysis of REACHE learner doctors demonstrates that RAS with medical training come to the UK from wherever there has been conflict, with vastly different personal circumstances and clinical experiences. With appropriate support, a high proportion of learners are successful in passing the required examinations on first attempt, and over half of learners achieve registration with the GMC and enter clinical practice. This is despite often having been out of medical practice for several years and managing traumas and challenges associated with displacement and restarting life in a new country. Alumni of the programme tend to practise within the geographical area that they were trained, with some also supporting more regional and rural areas across the UK.

There are several advantages in supporting RAS doctors in entering clinical practice within host countries. It supports professional identity retention, which in turn has a positive impact on mental health^16 17^; provides understaffed health workforces with clinicians who have more experience, are more cost-effective and quicker to train compared with UK-trained undergraduate medical students^5^; and reduces government expenditure on funded accommodation and financial assistance by allowing these RAS doctors to regain financial independence.^18^ These RAS doctors also have higher clinical examination pass rates and demonstrate higher retention within the health workforce compared with UK-trained undergraduate medical students^919^,^21^ and contribute to a diverse workforce with greater lived experiences and capacity to support RAS populations.^22^ Other host countries within Europe have garnered similar benefits, such as Türkiye’s programme to train Syrian refugee doctors and nurses to support their large Syrian refugee population.^23^

The first barrier (chronologically) in using these skilled professionals is the lack of any form of identification process of RAS with medical qualifications on entry to the UK. Therefore, they are not signposted or placed close to a training centre, such as REACHE.^5^ The long average duration between arriving in the UK and contacting REACHE and the propensity for word of mouth as the most common reason for contact further demonstrate this need for centrally organised signposting of refugees to appropriate NHS-funded education centres, as clinical relevance deteriorates over time when not practising.^16^ Furthermore, reliance on online searches for identifying RAS HCPs is limited by digital literacy barriers and confusion related to differences in technology use, language and terminology.^24^

Additional policy and process challenges include the limited rights for asylum seekers to undertake paid work within the UK as opposed to those who have been granted refugee status^3 25^ and removal of doctors from the ‘Immigration Salary List’, which limits sponsorship opportunities.^26^ Processing asylum-seeker documentation can take several years, compounding the stress experienced during forced relocation, limiting financial security and capacity to study and making registration success less likely, as demonstrated by the difference in this study among RAS. Removal of such legal limitations, in addition to reducing these time-related pressures on asylum seekers, is expected to save upwards of £6bn in government expenditure in addition to increased tax revenue of over £1bn.^23^

Funding barriers also remain an ongoing issue for such programmes, with the recent announcement of the dissolution of NHS England (the body responsible for funding UK RAS training programmes), an example of concerns on securing recurrent funding.^27^ With nearly one-fifth of all-time REACHE learners currently active in the programme, this rapid expansion necessitates commensurate resources to remain effective. The propensity for alumni to practise within their geographical region of training, alongside a large number of RAS living across other regions of the UK,^28^ demonstrates the need for an expanded offer nationally and increased utilisation of digital and remote resources and access to clinical placements. There is also the case, as in Germany, for ensuring that programmes such as REACHE that train RAS medical doctors and nurses with national monies invested in their training should be recognised as ‘home graduates’ within the context of employment and foundational jobs and not viewed as international medical graduates. This would ensure their initial placements and jobs would support them well and provide the security of long-term medical practice in the UK. Within the UK, a new programme (supported by REACHE) has been established in Belfast with the intention to provide Northern Ireland with another source of trained medical professionals to manage the health workforce shortage.^29^

There are strengths and limitations of this research to consider when reviewing the results and applying them to external contexts. The data have been sourced from reliable internal REACHE documentation over a prolonged period demonstrating these sustained positive outcomes, as well as from the GMC that contains up-to-date registration data. The large sample size and diversity of learners also indicate broader generalisability of the findings across countries of origin, differing family commitments and entry pathways. However, REACHE learners may be RAS who have more capacity to invest time in learning and achieving registration and may not necessarily reflect the broader population of RAS with healthcare experience. Also, some learners may take examinations prior to joining the programme or may choose not to disclose additional attempts to the programme, potentially contributing to an inaccurate first attempt pass rate. Finally, the restriction to quantitative data prevents insights into reasons behind the success or failure of learners within the programme as well as the programme itself.

## Conclusions

Global issues enforce the continued mass forced displacement of people including HCPs. Host countries can use these skilled individuals and concurrently allow them to maintain their professional abilities, which can then be used to rebuild their nations post-conflicts. RAS HCPs experience unique challenges and require tailored, holistic, educational, placement and employment supports in achieving registration and practising clinically. Training centres such as REACHE currently receive non-recurrent and variable funding yet provide tangible benefits for individual RAS doctors, their dependents and the health workforce and government expenditure. Acknowledging and including RAS HCPs in national medical workforce strategies is also necessary. Improved signposting from governmental organisations, such as the Home Office, to identify skilled HCPs and creating the mechanisms to support their entry to the NHS, such as using a commonsense approach to policy and barriers in law such as the Immigration Salary List (July 2024), will enhance these benefits for all parties.

## Supplementary material

10.1136/bmjopen-2025-105550 online supplemental file 1

## Data Availability

Data are available upon reasonable request.
