# Supplementary material for: Training refugee and asylum-seeking doctors: a cohort study of the UK REACHE programme
Source: BMJ Open. 2025 Nov 4;15(11):e105550. doi: 10.1136/bmjopen-2025-105550 (PMC12587900; doi:10.1136/bmjopen-2025-105550)
Supplement: online supplemental file 1 [file bmjopen-15-11-s001.docx]

**Appendix 1**. Specific specialties held by alumni of the REACHE programme.

1 x Anaesthetics

1 x Anaesthetics & Intensive Care Medicine

1 x Child and Adolescent Psychiatry

1 x Clinical Radiology

1 x Emergency Medicine

4 x General Internal Medicine & Acute Internal Medicine

3 x General Internal Medicine & Endocrinology and Diabetes Medicine

2 x General Internal Medicine & Respiratory Medicine

1 x General Internal Medicine & Gastroenterology

1 x General Internal Medicine & Renal Medicine

49 x General Practitioner

1 x General Practitioner & General Internal Medicine & Geriatric Medicine

2 x General Psychiatry

1 x General Surgery

3 x Histopathology

3 x Obstetrics and Gynaecology

3 x Old Age Psychiatry

1 x Plastic Surgery

1 x Rehab Psychiatry

1 x Otolaryngology

1 x Rheumatology

**Appendix 2**. List of countries of origin of REACHE learner doctors.

| Country of Origin | | Frequency | Percent |
| --- | --- | --- | --- |
|  | Afghanistan | 56 | 9.2 |
|  | Algeria | 1 | .2 |
|  | Armenia | 2 | .3 |
|  | Azerbaijan | 2 | .3 |
|  | Bangladesh | 4 | .7 |
|  | Belarus | 1 | .2 |
|  | Burundi | 4 | .7 |
|  | Chad | 2 | .3 |
|  | Congo | 3 | .5 |
|  | Democratic Republic of the Congo | 15 | 2.5 |
|  | Egypt | 15 | 2.5 |
|  | El Salvador | 2 | .3 |
|  | Eritrea | 7 | 1.2 |
|  | Ethiopia | 5 | .8 |
|  | Georgia | 3 | .5 |
|  | Guinea | 1 | .2 |
|  | Honduras | 2 | .3 |
|  | India | 1 | .2 |
|  | Indonesia | 1 | .2 |
|  | Iran | 31 | 5.1 |
|  | Iraq | 104 | 17.1 |
|  | Israel | 1 | .2 |
|  | Jordan | 2 | .3 |
|  | Kuwait | 1 | .2 |
|  | Lebanon | 2 | .3 |
|  | Libya | 47 | 7.7 |
|  | Lithuania | 1 | .2 |
|  | Myanmar | 9 | 1.5 |
|  | Nepal | 1 | .2 |
|  | Netherlands | 1 | .2 |
|  | Nigeria | 5 | .8 |
|  | Pakistan | 22 | 3.6 |
|  | Palestine | 7 | 1.2 |
|  | Russian Federation | 8 | 1.3 |
|  | Saudi Arabia | 5 | .8 |
|  | Senegal | 1 | .2 |
|  | Serbia | 1 | .2 |
|  | Somalia | 14 | 2.3 |
|  | Sri Lanka | 7 | 1.2 |
|  | Stateless | 1 | .2 |
|  | Sudan | 74 | 12.2 |
|  | Syria | 77 | 12.7 |
|  | Turkey | 9 | 1.5 |
|  | Ukraine | 18 | 3.0 |
|  | United Kingdom | 2 | .3 |
|  | Yemen | 13 | 2.1 |
|  | Zambia | 1 | .2 |
|  | Zimbabwe | 9 | 1.5 |
|  | Total | 601 | 99.0 |
|  | Missing Data | 6 | 1.0 |

**Appendix 3**. Numbers of REACHE alumni practicing in each region of the UK.

| England (North West) | 170 | |
| --- | --- | --- |
| England (North East and Yorkshire) | | 33 |
| England (Midlands) | 23 | |
| England (South West) | 4 | |
| England (East of England) | 14 | |
| England (London) | 25 | |
| England (South East) | 14 | |
| Scotland | 5 | |
| Wales | 22 | |
| Northern Ireland | 3 | |
