# Supplementary material for: Training refugee and asylum-seeking doctors: a cohort study of the UK REACHE programme
Source: BMJ Open. 2025 Nov 4;15(11):e105550. doi: 10.1136/bmjopen-2025-105550 (PMC12587900; doi:10.1136/bmjopen-2025-105550)
Supplement: Supplementary file 2 [file bmjopen-2025-105550.reviewer_comments.pdf]

PEER REVIEW HISTORY

BMJ Open publishes all reviews undertaken for accepted manuscripts. Reviewers are asked to complete a checklist review form and are provided with free text boxes to elaborate on their assessment. These free text comments are reproduced below.

ARTICLE DETAILS

Title (Provisional)

Cohort study of the UK REACHE programme: training refugee and asylum-seeking doctors

Authors

Drovandi, Aaron; Barrett, Samuel; Kallingal, Jouher; Awan, Aisha

---

VERSION 1 - REVIEW

**Reviewer**	**1**
**Name**	**Matsumoto, Monica**
**Affiliation**	**University of Chicago, Pritzker School of Medicine**
**Date**	**28-Jul-2025**
**COI**	**None**

Overall the manuscript is very well written and covers a unique program with terrific results. There are a few minor comments that can be addressed to strengthen the manuscript and clarify some questions.

Abstract:

- Conclusion does not accurately reflect the study results and is too far reaching – please adjust to focus on the study itself rather than future areas of focus. For example, was any analysis performed on reducing government expenditures?

Methods

- Did participants themselves provide consent?

- Are not all analyses technically "retrospective"? The authors can just define this as a prospective cohort study unless there is something specific that qualifies this as retrospective.

- Do the authors have any idea how many RAS HCPs were eligible for REACHE versus how many actually applied?

Results

- Do the authors have any qualitative data about participants' experiences with and feedback about the REACHE programme, or any other sort of qualitative follow-up that could provide more details on the programme alumni? This would provide interesting details to the manuscript, such as any programmatic feedback, satisfaction, and areas of improvement.

Discussion

- The limitations section should focus exclusively on limitations. The strengths can be discussed elsewhere, if at all.

- Another limitation is the limited conclusions that can be made due to lack of in-depth data collected. In depth interviews gathering more details on individual and collective experiences would provide more depth.

- The authors provide some financial arguments to support training RAS doctors, including the investments needed to train UK medical students. However, one should be cautious with any sort of cost-benefit proposal unless one is formally performed.

**Reviewer**	**2**
**Name**	**McGrail, Matthew R.**
**Affiliation**	**Univ Queensland, Rural Clinical School**
**Date**	**07-Aug-2025**
**COI**	**None**

This study evaluates 20+ years of activity and outcomes of the REACHE program. I think this is an important study to complete (and publish), but I found a number of key pieces of information were missing.

Firstly, the authors somewhat mislead in that the Intro and Methods refer to HCPs broadly (including the study Aim), but from the Results it suddenly switches to only Doctors. Exclusion of the other health professionals should be signalled / clear much earlier (outside of the title).

Being an evaluation of the REACHE program, it is really important to provide sufficient details about the program – currently it is greatly lacking. Figure 1 provides a brief structure (a few dot points), but that's all. There is a short paragraph towards the end of the Results (why is it there?), but more info is needed. It is really unclear how the support is provided, in what format. Is it 100% in-person? Does it have a fixed structure (e.g. 'semesters'), or can people join at any time? I suggest adding a significant section, perhaps with an accompanying 'Box', which more fully describes all components of the program.

I'm not based in the UK, so I'm less familiar with their local medical training and registration. In particular, I was quite confused with what these doctors can/can't do. They state a median 1.3 years from entering UK to joining the program. Please add range distribution info to this

(min, max, IQR). Also, please clarify their status during this period – are any able to practice medicine (in any capacity), or not? Then, they find a median 25 months in the program – again, please clarify if/how much they can practice medicine during this time. Finally, after completing the program – what is their status? i.e. what is their equivalent registration (FY1, FY2, etc)?

In the results, a series of pass rates and withdraw information is provided, which are important; being a program evaluation, these are most useful if they can be compared with alternative pathways. How do they compare to RAS without access to a program like REACHE? How do they compare to IMGs of similar demographics (but without the RAS status)? In the Discussion, they briefly allude to some pass rates and workforce retention being better than domestic (UK) med students. This should be further clarified, and preferably add other comparisons as above.

The distribution maps are interesting, but also hard to read. Perhaps an accompanying table of % in each of 5-6 geographic regions would greatly assist.

I note that 'only' ~80 with GMC registration have completed their specialty training. I'm assuming that this is strongly correlated with recency of their registration (i.e. not having enough time to have also completed their specialty training). Perhaps it'd be useful to further break this down by when they completed their GMC registration and if they've subsequently completed local specialty training. E.g. What proportion of those with GMC registration ~8 years ago (e.g. pre 2016) completed specialty training? This will provide a better perspective of how many complete this next step.

The authors make an 'interesting' suggestion in the discussion, that they be recognised as 'home graduates' rather than IMGs. Not sure I fully agree, but I do agree from literature I've seen which suggests these types of IMGs are seeking a more settled / stable life and thus any policy which can support their retention in this way is desirable.

One point at the end of the Discussion and repeated in the strengths/limitations summary relates to whether the program participants are 'biased', suggesting they may be those with more time / availability. I'm not really convinced this is likely to be a big factor; their motivation to return to clinical practice is probably the key factor.

VERSION 1 - AUTHOR RESPONSE

Reviewer 1:

Overall the manuscript is very well written and covers a unique program with terrific results. There are a few minor comments that can be addressed to strengthen the manuscript and clarify some questions.

Response: The authors appreciate the reviewers' overall positive response to our manuscript, and for their comments on how we might improve its quality.

1. <u>Abstract:</u>
   a. Conclusion does not accurately reflect the study results and is too far reaching – please adjust to focus on the study itself rather than future areas of focus. For example, was any analysis performed on reducing government expenditures?
      <u>Response:</u> We have modified the conclusion subsection of the abstract to better align with the key findings. The rewritten part of this section now reads: "Retraining programmes such as REACHE can effectively support RAS doctor requalification, providing the UK medical workforce with experienced professionals."

2. <u>Methods:</u>
   a. Did participants themselves provide consent?
      <u>Response:</u> Thank-you for identifying this omission from the original submission. Yes, each participant provides consent to have their de-identified data to be included in research as part of their application and induction processes when joining REACHE, prior commencing any learning within the programme. We have added in the following text to reflect this: "All REACHE learners provide consent to have their data stored in the REACHE database and for anonymised data to be used for research and grant funding purposes."

   b. Are not all analyses technically "retrospective"? The authors can just define this as a prospective cohort study unless there is something specific that qualifies this as retrospective.
      <u>Response:</u> Whilst the cohort itself has had its data collected prospectively, this specific use of the data was not planned from the outset of the data collection (>20 years ago). We therefore prefer to be transparent in that we are conducting analysis of data that was collected prior the design of this specific study. The authors have used this distinction in previous research and hope this satisfies the reviewers' comment.

   c. Do the authors have any idea how many RAS HCPs were eligible for REACHE versus how many actually applied?
      <u>Response:</u> Yes this data has been collected over the past few years, though this was not the case in the earlier years of the programme. For example, for the 3-year period from mid-2022 to mid-2025, there have been 129 learners admitted to the programme compared to 581 who did not progress to this stage. The most common reasons include simply not maintaining contact after inquiring, live proximal to an alternate training programme which we refer them to, an issue with confirming their previous qualifications and experience as a doctor, or had already been out of practice for more than 10 years before entering the UK. We believe these details are out of scope of the paper and should remain omitted, however we plan to publish further research relating to the programme and will include such details highlighting the scope of the training needs of refugee and asylum seeking doctors.

3. <u>Results:</u>
   a. Do the authors have any qualitative data about participants' experiences with and feedback about the REACHE programme, or any other sort of qualitative follow-up that could provide more details on the programme alumni? This would provide interesting details to the manuscript, such as any programmatic feedback, satisfaction, and areas of improvement.
      <u>Response:</u> This study is strictly a quantitative one, focussing on the objective outputs of the REACHE programme since its inception. The authors (through previous experience) believe that attempting to integrate rigorous qualitative data would make it unwieldy as a single article and dilute key findings. However, the authorship team are undertaking separate research projects including the financial aspects of RAS doctor training, and qualitative data on the experiences of RAS doctors, and will cite this article within them to demonstrate the

multifaceted elements of how the programme supports refugees and asylum seekers. We appreciate the reviewers' interest in the qualitative aspects of RAS doctor retraining.

4. Discussion
   a. The limitations section should focus exclusively on limitations. The strengths can be discussed elsewhere, if at all.
   Response: It is common practice for both strengths and limitations of research to be presented together to frame reliability and wider applicability of the findings, and is part of the instructions to authors for papers in BMJ Open.

   b. Another limitation is the limited conclusions that can be made due to lack of in-depth data collected. In depth interviews gathering more details on individual and collective experiences would provide more depth.
   Response: The authors agree with this suggestion and have added the following text into the final paragraph of the discussion with the other limitations: "Finally, the restriction to quantitative data prevents insights into reasons behind success or failure of learners within the programme as well as the programme itself."

   c. The authors provide some financial arguments to support training RAS doctors, including the investments needed to train UK medical students. However, one should be cautious with any sort of cost-benefit proposal unless one is formally performed.
   Response: The authors have been cautious to only refer to the potential financial benefits of RAS doctor training within the discussion with appropriate references (including our previous work published in the BMJ which gives broad-stroke figures relating to costs of training UK undergraduate medical students compared to RAS doctors) – having taken the mention of finances out of the abstract we hope this satisfied the reviewers' comment.

Reviewer 2:

This study evaluates 20+ years of activity and outcomes of the REACHE program. I think this is an important study to complete (and publish), but I found a number of key pieces of information were missing.
Response: The authors thank the reviewer for their balanced opinion of the manuscript, and welcome their recommendations below, which we have attended to.

1. Firstly, the authors somewhat mislead in that the Intro and Methods refer to HCPs broadly (including the study Aim), but from the Results it suddenly switches to only Doctors. Exclusion of the other health professionals should be signalled / clear much earlier (outside of the title).
   Response: The authors thank the reviewer for highlighting this inconsistency in scope. The intention was to indicate we are understating the benefits of REACHE through focusing on doctors as the bulk of our cohort, though acknowledge it may be misleading. We have modified the last paragraph of the introduction and relevant sections within the methods to refer to 'RAS doctors' rather than 'RAS HCP' to prevent this misinterpretation.

2. Being an evaluation of the REACHE program, it is really important to provide sufficient details about the program – currently it is greatly lacking. Figure 1 provides a brief structure (a few dot points), but that's all. There is a short paragraph towards the end of the Results (why is it there?), but more info is needed. It is really unclear how the support is provided, in what format. Is it 100% in-person? Does it have a fixed structure (e.g. 'semesters'), or can people join at any time? I suggest adding a significant section, perhaps with an accompanying 'Box', which more fully describes all components of the program.

Response: Thank-you for raising this point. We have historically been cautious in 'giving too much away' regarding the structure of the programme due to concerns that other organisations (particularly those that are for profit) would adopt the programme and draw away what limited funding we have. However, we acknowledge the necessity for providing enough details that readers can understand the programme sufficiently to apply the evaluation findings. We have inserted a new paragraph in the introduction alongside the first reference to Figure 1, which provides further details of the programme, which reads: "**Figure 1** illustrates the individual components and directionality of learner journeys through the programme. On entry into the programme, learners are provided with continual pastoral support on a range of personal issues that support settling into the UK, as well as support in securing 'lower grade' clinical employment for improved financial stability while learning. The educational programme is run across three semesters in a hybrid format, taught and supported by clinical tutors and senior lecturers from the University of Manchester, and language teachers at a hospital-based education centre. English-language training and exam preparation are provided at two levels according to English proficiency at entry, including practice conversations and other short activities, daily questions and practice examinations. The clinical curriculum is based on the requirements for the General Medical Council (GMC) Medical Licencing Exam (MLA). Learners also complete a reflective portfolio, attend regular whole day 'integrated communication and clinical skills' days covering topics to help adapt to life and work in the UK, clinical role plays, clinical reasoning, opportunities for research and audit, and simulation suite sessions alongside their weekday teaching."

3. I'm not based in the UK, so I'm less familiar with their local medical training and registration. In particular, I was quite confused with what these doctors can/can't do. They state a median 1.3 years from entering UK to joining the program. Please add range distribution info to this (min, max, IQR). Also, please clarify their status during this period – are any able to practice medicine (in any capacity), or not? Then, they find a median 25 months in the program – again, please clarify if/how much they can practice medicine during this time. Finally, after completing the program – what is their status? i.e. what is their equivalent registration (FY1, FY2, etc)?
Response: Thank-you for these suggestions which will make the article findings easier to interpret for international readers. We have added the following distribution data relating to UK entry and joining the programme: "The median time for doctor learners from entering the UK to initial contact with REACHE was 1.3 years (interquartile range of 6 months to 3 years, a minimum of 2 weeks and maximum of 15.6 years)". We have also expanded on pre and post-programme restrictions on practice. In the introduction (relevant to all healthcare professions) we have added: "RAS HCPs entering the UK are unable to practice their profession. Restrictions result in their working in support roles such as healthcare assistants or phlebotomists, often requiring additional training, despite their existing expertise and qualifications." Toward the end of the introduction we have also added (specific to doctors) the following: "On achieving registration, RAS doctors may work clinically in the UK, entering at a 'Foundation Year 2' level, though this may not be at their highest level of qualification."

4. In the results, a series of pass rates and withdraw information is provided, which are important; being a program evaluation, these are most useful if they can be compared with alternative pathways. How do they compare to RAS without access to a program like REACHE? How do they compare to IMGs of similar demographics (but without the RAS status)? In the Discussion, they briefly allude to some pass rates and workforce retention being better than domestic (UK) med students. This should be further clarified, and preferably add other comparisons as above.
Response: Unfortunately this data does not yet exist within the UK making comparisons or assumptions impossible. The 'closest' data available is the General Medical Council reports on the number of IMGs registering, though this includes refugees and asylum seekers within this

count and it is not possible to separate them. Regarding comparisons to domestic students, to avoid self-plagiarism we have referred to our previous BMJ paper which provides more detail on comparisons relating to workforce retention and pass rates.

5. The distribution maps are interesting, but also hard to read. Perhaps an accompanying table of % in each of 5-6 geographic regions would greatly assist.
   Response: We have added a short table into the appendix which indicates the number of alumni registered within each region of England, as well as the smaller numbers within Scotland, Wales and Northern Ireland.

6. I note that 'only' ~80 with GMC registration have completed their specialty training. I'm assuming that this is strongly correlated with recency of their registration (i.e. not having enough time to have also completed their specialty training). Perhaps it'd be useful to further break this down by when they completed their GMC registration and if they've subsequently completed local specialty training. E.g. What proportion of those with GMC registration ~8 years ago (e.g. pre 2016) completed specialty training? This will provide a better perspective of how many complete this next step.
   Response: The authors appreciate this suggestion and can confirm that it is a combination of recency of registration and the expanding nature of the REACHE programme which has limited the total number of alumni who have completed specialty training (>25% of doctors). Under recent changes in leadership, the programme has rapidly expanded the number of RAS doctors it supports, with nearly 1/5 of all doctors ever admitted still within the programme. This is reflected in the timing of achieving specialty registration: 1 in 2008, 1 in 2009, 4 in 2010, 4 in 2011, 3 in 2012, 7 in 2013, 6 in 2014, 6 in 2015, 10 in 2016, 9 in 2017, 6 in 2018, 6 in 2019, 3 in 2020, 3 in 2021, 5 in 2022 [consider COVID for these years], 6 in 2023 and 2 in 2024.

7. The authors make an 'interesting' suggestion in the discussion, that they be recognised as 'home graduates' rather than IMGs. Not sure I fully agree, but I do agree from literature I've seen which suggests these types of IMGs are seeking a more settled / stable life and thus any policy which can support their retention in this way is desirable.
   Response: The argument for recognition as home students stems both from a policy standpoint as well as from a training standpoint. For these learners (compared to other international graduates), the UK has invested resources into their training and development, including clinical placements which makes them better prepared for UK practice.

8. One point at the end of the Discussion and repeated in the strengths/limitations summary relates to whether the program participants are 'biased', suggesting they may be those with more time / availability. I'm not really convinced this is likely to be a big factor; their motivation to return to clinical practice is probably the key factor.
   Response: We have been careful to state that this is a possible factor rather than a definitive factor. Our experience with learners and applicants anecdotally has suggested that the time investment into training and registration may not be feasible for those with families who have relocated and are instead required to work full-time, often across multiple 'lower-level' jobs.

VERSION 2 - REVIEW

Reviewer **2**

Name **McGrail, Matthew R.**

Affiliation **Univ Queensland, Rural Clinical School**

Date **08-Oct-2025**

COI

All concerns have been adequately addressed
